# Comprehensive Analysis of Transcriptional Expression of hsa-mir-21 Predicted Target Genes and Immune Characteristics in Kidney Renal Clear Cell Carcinoma

**DOI:** 10.7150/ijms.73404

**Published:** 2022-08-21

**Authors:** Da-Ming Xu, Ming Li, Shu-Bin Lin, Zheng-Liang Yang, Teng-Yu Xu, Jin-Huan Yang, Jun Yin

**Affiliations:** 1 Department of Urological Surgery, Second Affiliated Hospital of Shantou University Medical College, Shantou, Guangdong, China.; 2 Department of Hematology, Second Affiliated Hospital of Shantou University Medical College, Shantou, Guangdong, China.; 3 Department of Clinical Laboratory Medicine, Second Affiliated Hospital of Shantou University Medical College, Shantou, Guangdong, China.

**Keywords:** Hsa-mir-21, Predicted Target Gene, Immune Characteristics, Transcription Factor, Prognosis, Prediction Model, Kidney Renal Clear Cell Carcinoma

## Abstract

**Background:** To uncover advanced prognosis biomarkers in patient with kidney renal clear cell carcinoma (KIRC), our study was the first to make a comprehensive analysis of hsa-mir-21 predicted target genes and explore the immune characteristics in KIRC.

**Methods:** In this study, the comprehensive analysis of hsa-mir-21 predicted target genes and immune characteristics in KIRC were analyzed via TIMER2.0, UALCAN, Metascape, Kaplan-Meier plotter, Human Protein Atlas, CancerSEA, JASPAR, GEPIA, R package: GSVA package (version 1.34.0) & immune infiltration algorithm (ssGSEA) and R package: RMS package (version 6.2-0) & SURVIVAL package (version 3.2-10).

**Results:** Up-transcriptional expressions of RP2, NFIA, SPRY1 were significantly associated with favorable prognosis in KIRC, whereas that of TGFBI was markedly significantly to unfavorable prognosis. Additionally, RP2, NFIA, SPRY1 and TGFBI were significantly relevant to the immune infiltration in KIRC. Finally, ZNF263 was a common predicted transcription factor of RP2, NFIA, SPRY1 and TGFBI, which can as an independent indicator for prognosis in KIRC patients.

**Conclusions:** Hsa-mir-21 predicted target genes (RP2, NFIA, SPRY1 and TGFBI) and the common transcription factor ZNF263 could be the advanced prognosis biomarkers in KIRC patients.

## Introduction

Renal cell carcinoma was a heterogeneous cancer originating from renal tubular epithelial cells and was one of the 10 most common cancers in the world 1,2. Kidney renal clear cell carcinoma (KIRC) was the most common subtype of renal cell carcinoma and was the leading cause of death in kidney cancer 3, followed by the papillary and chromophobe subtypes 4,5. About 60% of KIRC patients present with localized renal masses, but one-third of patients with localized KIRC eventually end up with metastatic disease, which needed a systemic treatment and was related to the poor prognosis 6-8. The incidence of KIRC increased significantly with age and was higher in males than in females 1. In addition to obesity, hypertension and smoking, some kidney related diseases, such as chronic kidney disease, renal cystic disease, as well as kidney transplantation and hemodialysis have all been shown to be risk factors for kidney cancer 1. Currently, genetic factors have also been a focus of KIRC research, with a number of kidney cancer risk genes, including VHL(3p25-26), BAP1(3p21), SDHB(1p36), SDHC(1q23) and SDHD(11q23) genes, which were reported to implicate in the development of KIRC 9-12. In addition, there were three hereditary syndromes associated with KIRC including von Hippel-Lindau disease, BAP1 mutant disease and SDH-associated kidney cancer 1. Therefore, we provide insights into the KIRC genetic risk to discover new prognostic biomarkers.

MicroRNAs (miRNAs) were the post-transcriptional regulators, which can play an important role in cancers' occurrence and development by negatively regulating the expression of target genes 13. Almost all tumors involved different types of miRNA expression and their predicted target genes expression, and the expression of miRNA predicted target genes were usually in up-regulated, down-regulated or basically unchanged state in tumors, suggesting that some miRNA predicted target genes may become tumor prognostic biomarkers. It has been found that hsa-mir-21 predicted target genes were involved in the occurrence and development processes in different tumors in recent studies 14, 15. Amirfallah et al. found that high hsa-mir-21 levels were related to poor survival and lymph node positivity in breast cancer patients, suggesting that hsa-mir-21 was a marker of poor prognosis for breast cancer 16. Wang et al. reported that the higher level of hsa-mir-21 in gastric cancer tissues were associated with the lower overall survival rate of patients 17. Additionally, hsa-mir-21 has been reported to have 95 targets associated with nasopharyngeal carcinoma 18.

As the few studies on the molecular basis of KIRC, and we believe that hsa-mir-21 and its predicted target genes have not been reported previously in patient with KIRC. To reveal potential prognostic biomarkers in KIRC, the role of hsa-mir-21 and its over-expressed predicted target genes in KIRC patients were first investigated in our study.

## Materials and Methods

The prediction of hsa-mir-21 target genes are selected based on the integration of three databases, including TargetScan (https://www.targetscan.org), microRNA.org (https://ngdc.cncb.ac.cn/databasecommons) and miRDB (http://mirdb.org). To assess the prognostic significance of hsa-mir-21 predicted target genes and immune characteristics in KIRC patients, relevant clinical characteristic data and transcriptional expression of hsa-mir-21 predicted target genes were analyzed by UALCAN. UALCAN (http://ualcan.path.uab.edu) is a publicly oncoomics database for the prediction and identification of biomarkers or potential target genes. The pan cancer analysis of hsa-mir-21 predicted target genes (RP2, NFIA, SPRY1 and TGFBI) and immune infiltration level in KIRC were analyzed by TIMER 2.0. TIMER 2.0 (http://timer.cistrome.org) is a database for the investigation of the association between immune infiltration and genetic or clinical characteristics. The KEGG and GO analysis of hsa-mir-21 predicted target genes (RP2, NFIA, SPRY1 and TGFBI) were analyzed by Metascape. Metascape (https://metascape.org) is a database that enables enrichment analysis of genes or proteins and construction of protein-protein interaction networks. The Overall Survival (OS) of hsa-mir-21 predicted target genes (RP2, NFIA, SPRY1 and TGFBI) were analyzed by Kaplan-Meier plotter. Kaplan-Meier plotter (http://www.kmplot.com) is a relational database that evaluates genes and survival parameters in tumors. The subcellular location, protein expression and single cell analysis of hsa-mir-21 predicted target genes (RP2, NFIA, SPRY1 and TGFBI) were analyzed by Human Protein Atlas. Human Protein Atlas (https://www.proteinatlas.org) is a proteomic and transcriptomic database that describes protein expression in tumor and normal tissues. The correlation of expression of hsa-mir-21 predicted target genes (RP2, NFIA, SPRY1 and TGFBI) with immune infiltration in KIRC were analyzed by R package: GSVA package (version 1.34.0) & immune infiltration algorithm (ssGSEA). The associated cancer functional states of hsa-mir-21 predicted target genes (RP2, NFIA, SPRY1 and TGFBI) were analyzed by CancerSEA. CancerSEA (http://biocc.hrbmu.edu.cn/CancerSEA/home.jsp) is a database that explores the functional heterogeneity of cancer cells based on single-cell sequencing technology. The Nomogram of KIRC containing variates (T stage, N stage, M stage, Age, Gender, RP2, NFIA, SPRY1, TGFBI) was analyzed by R package: RMS package (version 6.2-0) & SURVIVAL package (version 3.2-10). Transcription factor prediction and DNA base change of hsa-mir-21 predicted target genes (RP2, NFIA, SPRY1 and TGFBI) were analyzed by JASPAR. JASPAR (https://jaspar.genereg.net) is a database for predicting the binding regions of transcription factors to sequences. The disease free survival and overall survival analysis of ZNF263 in KIRC and the correlation between ZNF263 and hsa-mir-21 predicted target genes (RP2, NFIA, SPRY1 and TGFBI) were verified by GEPIA. GEPIA (http://gepia.cancer-pku.cn) is a database containing single gene analysis, cancer type analysis and multiple gene analysis.

## Results

### Up-expression of miRNAs in KIRC patients

**Figure 1:**
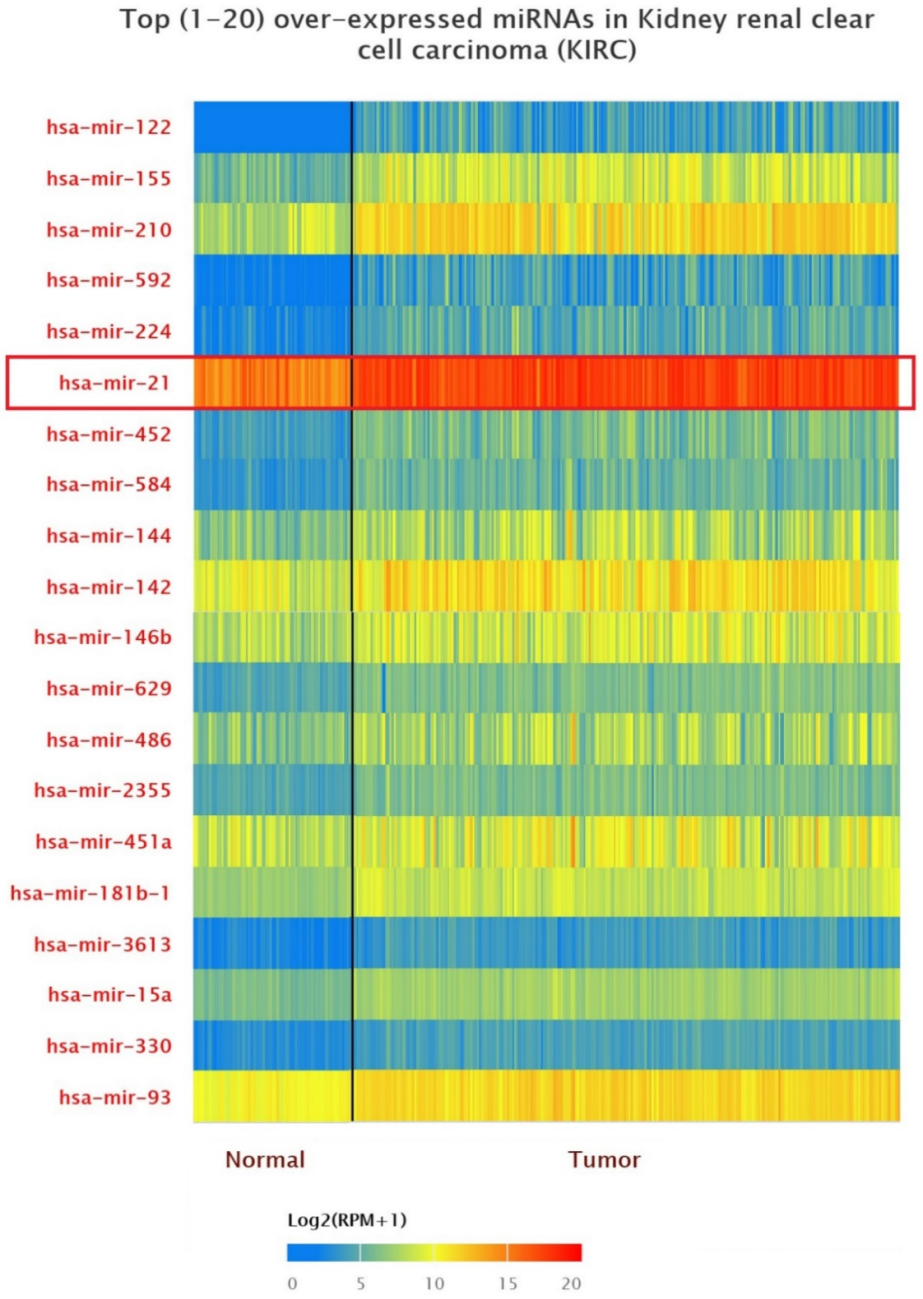
Top 20 over-expressed miRNAs in KIRC.

**Figure 2:**
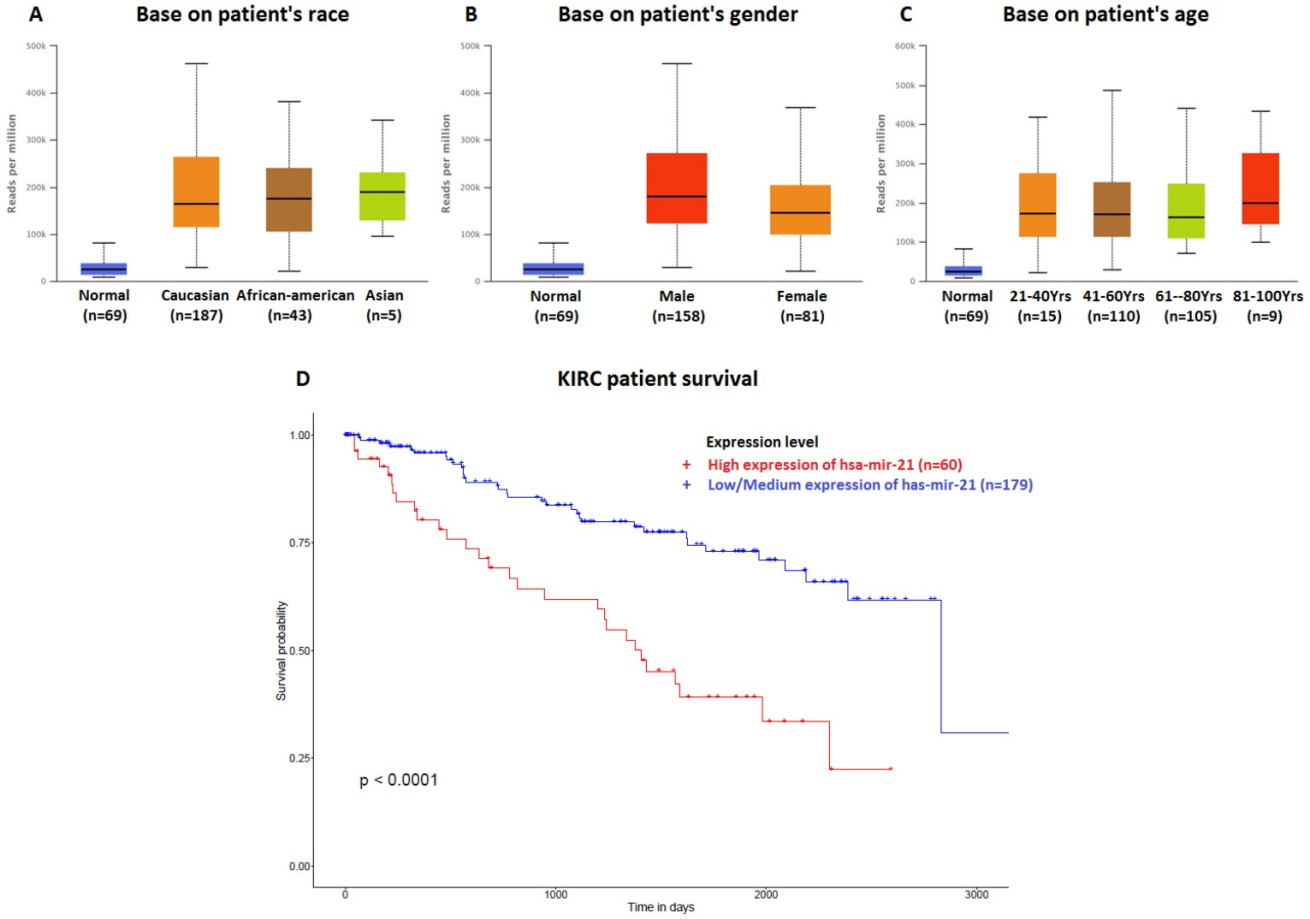
Hsa-mir-21 expression profile in KIRC based on patient's race, gender and age. Up-expression of hsa-mir-21 predicted target genes in KIRC patients.

The data of top (1-20) up-expressed miRNAs in KIRC patients were analyzed by TCGA (UALCAN) **(Figure 1)**. The hsa-mir-21 expression was markedly increased in KIRC patients (Normal-vs-Primary: *P*<0.001). Similarly, the hsa-mir-21 expression profile in KIRC based on patient's race, gender and age were analyzed by TCGA (UALCAN)** (Figure 2)**. Based on patient's race, the Normal-vs-Caucasian (*P*<0.001), Normal-vs-AfricanAmerican (*P<0.001*) and Normal-vs-Asian (*P*<0.05) had statistical significance **(Figure 2A)**. Based on patient's gender, the Normal-vs-Male (*P*<0.001), Normal-vs-Female (*P*<0.001) and Male-vs-Female (*P*<0.05) had statistical significance **(Figure 2B)**. Based on patient's age the Normal-vs-Age (21-40 Yrs) (*P*<0.001), Normal-vs-Age (41-60 Yrs) (*P*<0.001), Normal-vs-Age (61-80 Yrs) (*P*<0.001) and Normal-vs-Age (81-100 Yrs) (*P*<0.001) had statistical significance **(Figure 2C)**. In addition, there was significant difference in the effect of has-mir-21 expression level on KIRC patient survival **(Figure 2D)**. High expression was significant associated with unfavorable prognosis compared with low or medium expression (*P*<0.001).

**Figure 3:**
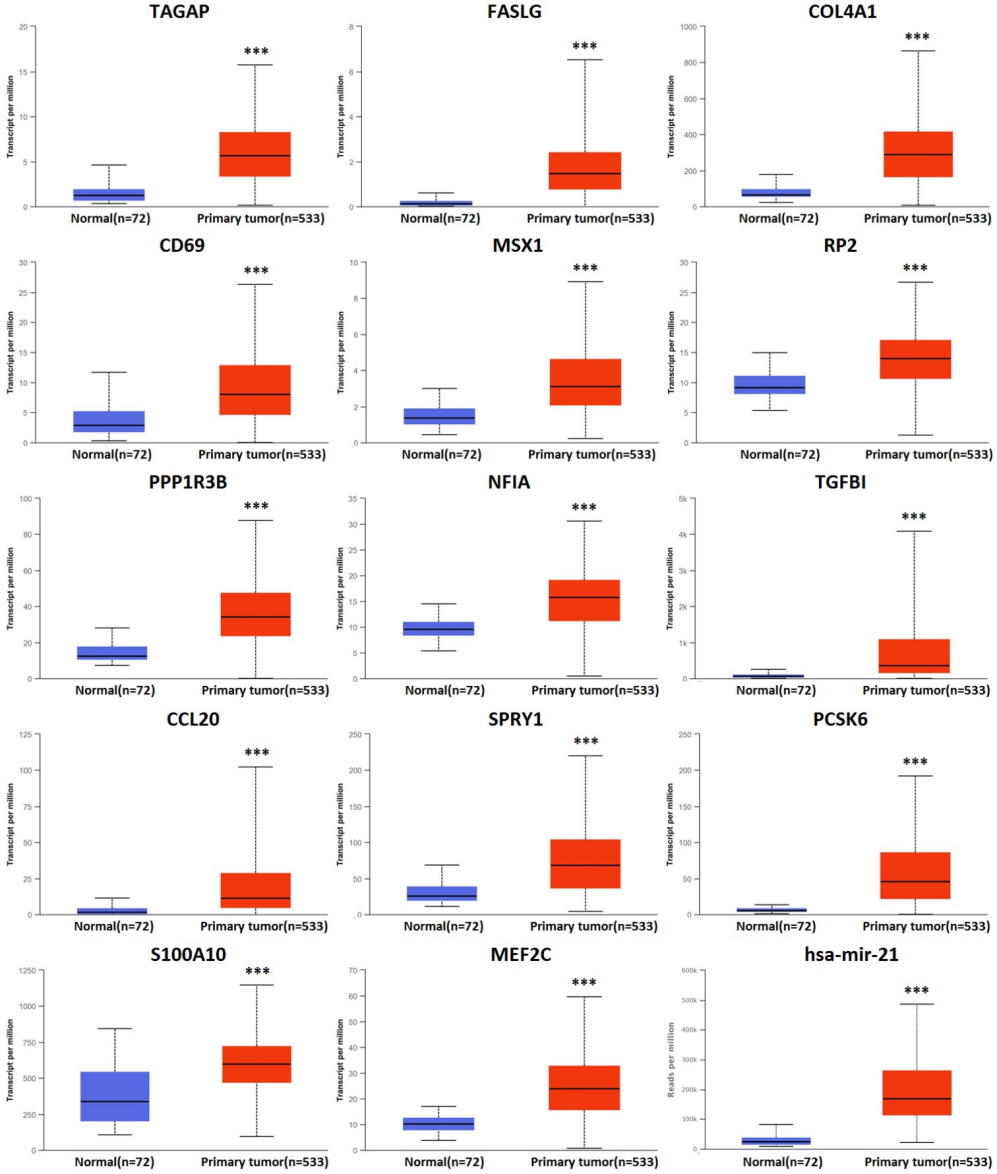
Transcriptional expression of hsa-mir-21 predicted target genes in KIRC (***: *P* < 0.001, **: *P* < 0.01, *: *P* < 0.05).

There were 158 hsa-mir-21 predicted genes in KIRC (Data from TargetScan, microRNA.org & miRDB), containing 14 cases of up-regulation, 40 cases of down-regulation and 104 cases of no change in KIRC **(Supplementary 1)**. 14 up-regulated hsa-mir-21 predicted genes in KIRC were selected to our study **(Table 1)**. The transcriptional expression of all up-regulated target genes in KIRC patients were markedly increased **(Figure 3)**.

### Predicted functional pathways of hsa-mir-21 up-expressed predicted target genes and other 30 gene partners

**Figure 4:**
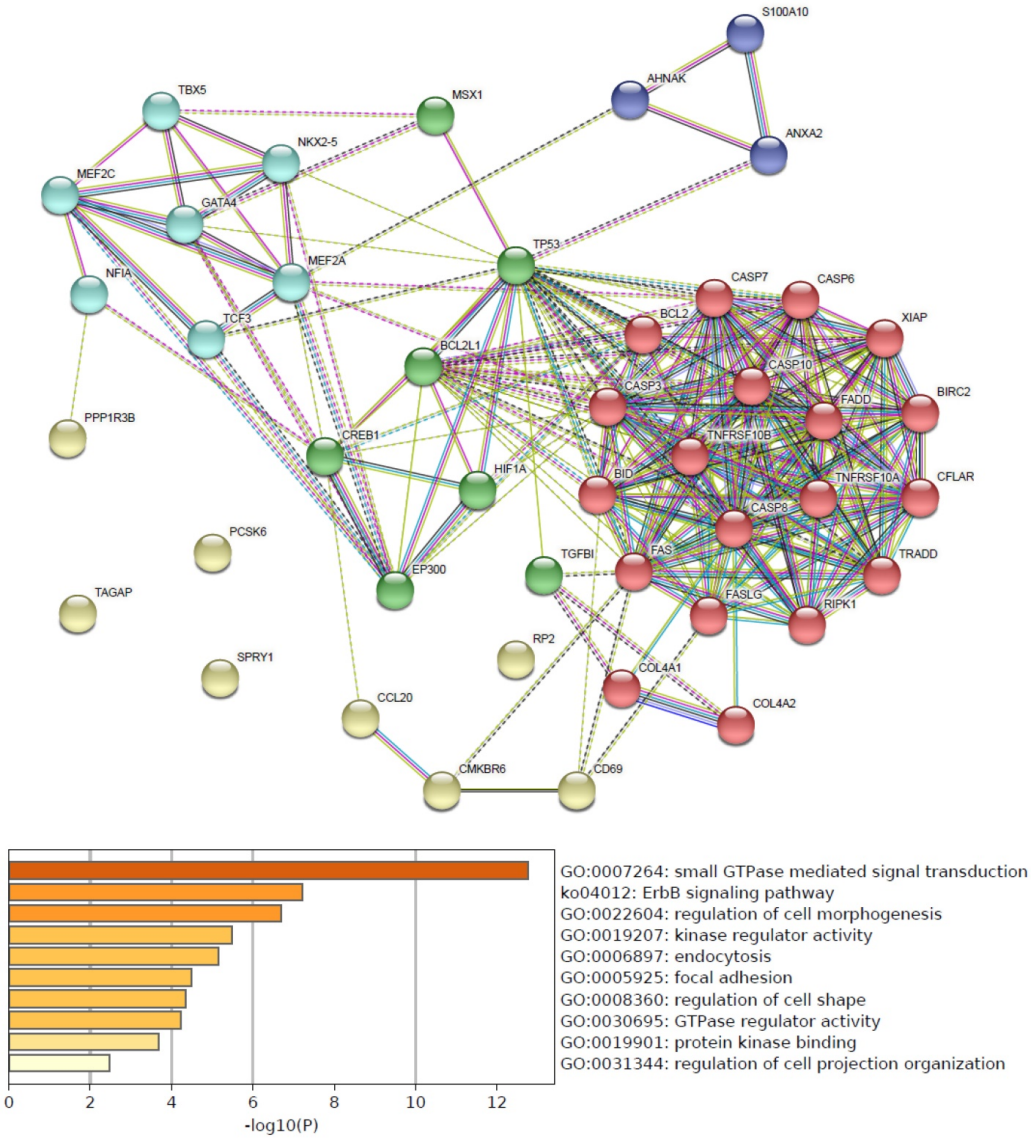
Predicted functional pathways of hsa-mir-21 up-expressed predicted target genes.

Functional pathways of hsa-mir-21 up-expressed predicted target genes and other 30 gene partners were analyzed by GO and KEGG (Metascape). As the functional pathways enrichment heatmap uncovered that GO:0007264 (small GTPase mediated signal transduction), ko04012 (ErbB signaling pathway), GO:0022604 (regulation of cell morphogenesis), GO:0019207 (kinase regulator activity), GO:0006897 (endocytosis), GO:0005925 (focal adhesion), GO:0008360 (regulation of cell shape), GO:0030695 (GTPase regulator activity), GO:0019901 (protein kinase binding) and GO:0031344 (regulation of cell projection organization) **(Figure 4)**.

### Pan cancer analysis and overall survival of mRNA expression of over-expressed hsa-mir-21 predicted target genes in KIRC

**Figure 5:**
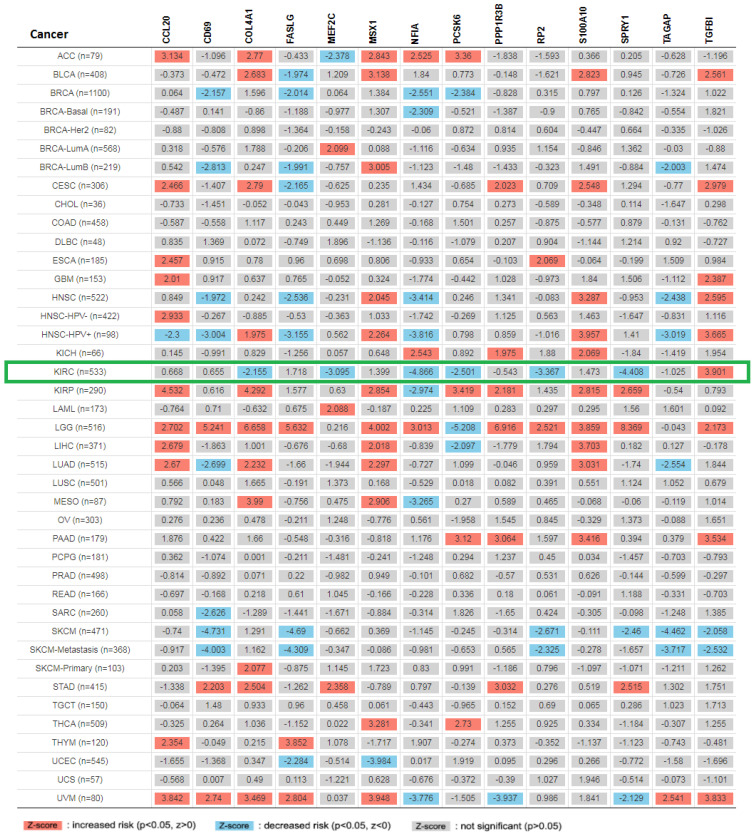
Expression of 14 up-expressed hsa-mir-21 predicted target genes in different cancer.

**Figure 6:**
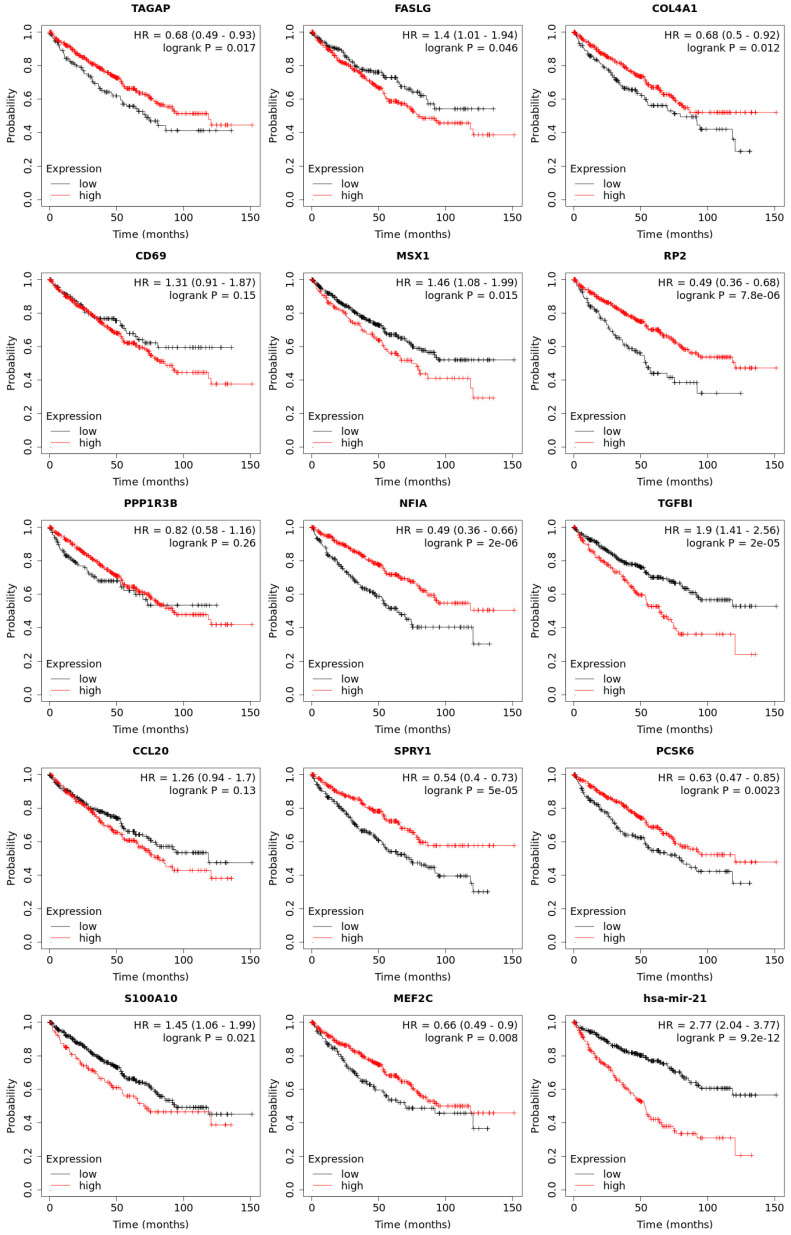
Overall survival of transcriptional expression of up-expressed hsa-mir-21 predicted target genes in KIRC patient.

Expression of 14 genes in different cancer were analyzed via TIMER, with red representing increased risk and blue representing reduced risk **(Figure 5)**. Overall survival of mRNA expression of over-expressed hsa-mir-21 predicted target genes were analyzed by Kaplan-Meier plotter **(Figure 6)**. The transcriptional expressions of TAGAP, FASLG, COL4A1, MSX1, RP2, NFIA, TGFBI, SPRY1, PCSK6, S100A10 and MEF2C were significantly relevant to KIRC prognosis. According to the Kaplan-Meier plotter analysis, TAGAP (HR=0.68, 95%CI: 0.49-0.93, *P*=0.017), COL4A1 (HR=0.68, 95%CI: 0.5-0.92, *P*=0.012), RP2 (HR=0.49, 95%CI: 0.36-0.68, *P*=7.8e-06), NFIA (HR=0.49, 95%CI: 0.36-0.66, *P*=2e-06), SPRY1 (HR=0.54, 95%CI: 0.4-0.73, *P*=5e-0.5), PCSK6 (HR=0.63, 95%CI: 0.47-0.85, *P*=0.0023) and MEF2C (HR=0.66, 95%CI: 0.49-0.9, *P*=0.008) were favorable for the prognosis of KIRC patients. FASLG (HR=1.4, 95%CI: 1.01-1.94, *P*=0.046), MSX1 (HR=1.46, 95%CI: 1.08-1.99, *P*=0.015), TGFBI (HR=1.9, 95%CI: 1.41-2.56,* P*=2e-0.5) and S100A10 (HR=1.45, 95%CI: 1.06-1.99, *P*=0.021) were unfavorable for the prognosis of KIRC patients. Among them, there were four genes with *P* values less than 0.001, namely RP2, NFIA, SPRY1 and TGFBI, which were the focus of our research. In addition, CD69 (HR=1.31, 95%CI: 0.91-1.87, *P*=0.15), PPP1R3B (HR=0.82, 95%CI: 0.58-1.16, *P*=0.26) and CCL20 (HR=1.26, 95%CI: 0.94-1.7, *P*=0.13) were no statistical significance in the prognosis of KIRC patients.

### Subcellular location and protein expression of hsa-mir-21 predicted target genes (RP2, NFIA, SPRY1 and TGFBI) in KIRC patients

**Figure 7:**
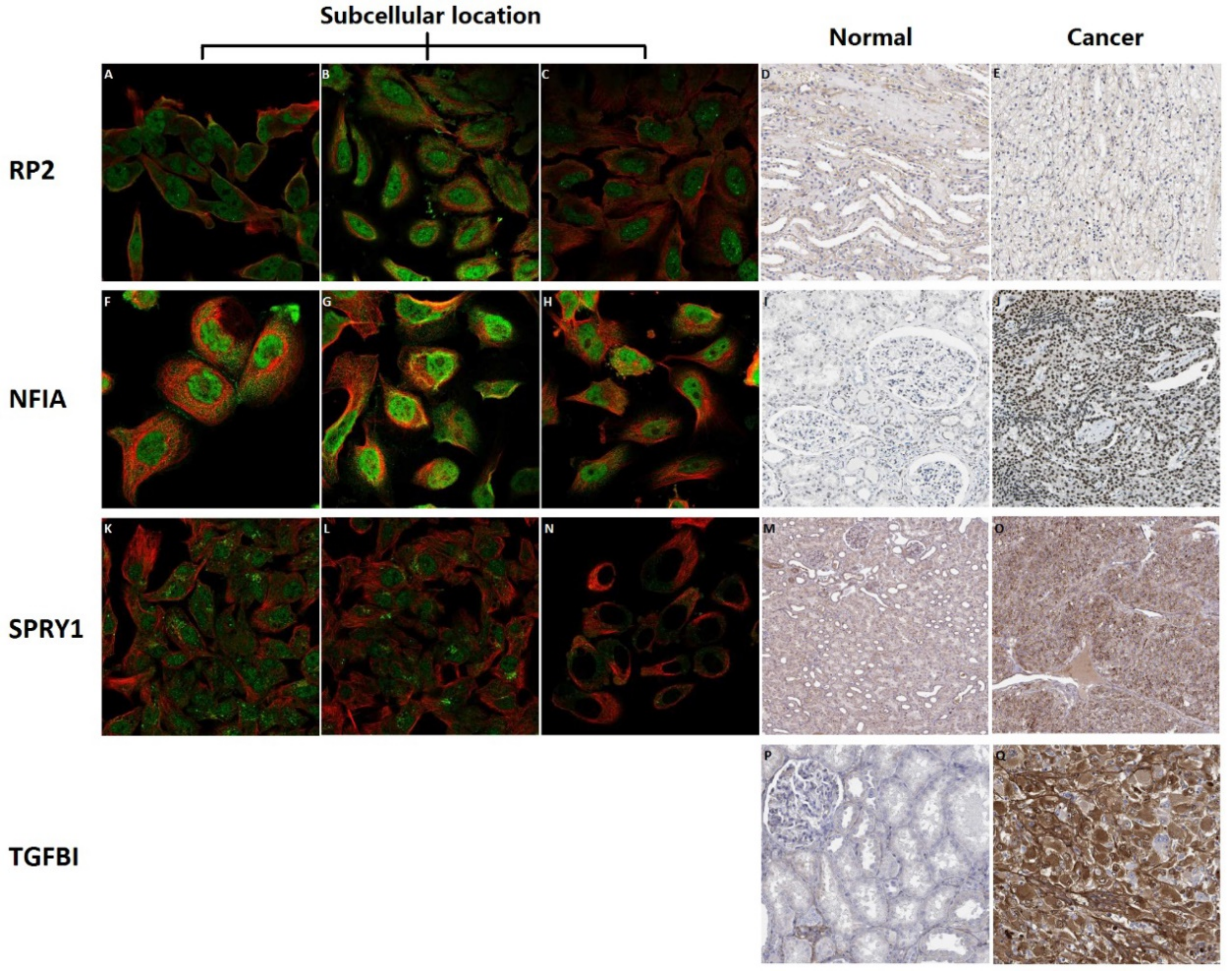
Subcellular location and protein expression of hsa-mir-21 predicted target genes (RP2, NFIA, SPRY1 and TGFBI) in KIRC.

Subcellular location and protein expression of hsa-mir-21 predicted target genes (RP2, NFIA, SPRY1 and TGFBI) in KIRC were analyzed by Human Protein Atlas** (Figure 7)**. RP2 mainly localized to the nucleoplasm and plasma membrane, and also localized to the nuclear bodies. NFIA mainly localized to the nucleoplasm. SPRY1 mainly localized to the cytosol and the Golgi apparatus, and also localized to the nucleoplasm. The protein expression of RP2 was nearly not detected in renal cancer, whereas low expression of that was found in normal kidney tissues. It is noteworthy that there was no absolute difference in the protein expression level of NFIA and SPRY1 between normal kidney tissues and kidney cancer. Additionally, different levels of protein expression intensity of TGFBI were observed in kidney cancer tissues but not detected in normal tissues.

### Association of transcriptional expression of hsa-mir-21 predicted target genes (RP2, NFIA, SPRY1 and TGFBI) and clinicopathological parameters in KIRC patients

**Figure 8:**
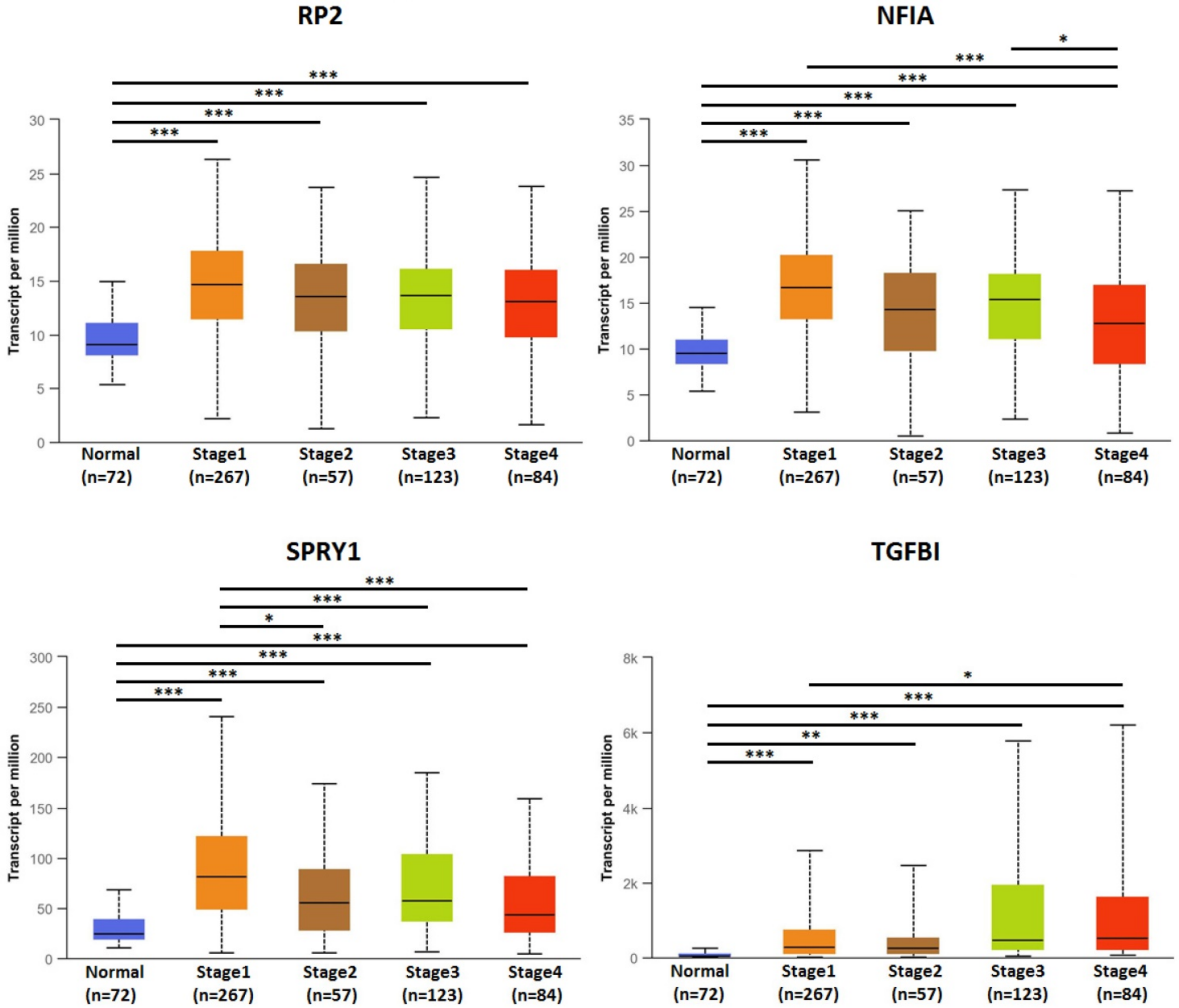
The association of transcriptional expression of hsa-mir-21 predicted target genes (RP2, NFIA, SPRY1 and TGFBI) and KIRC stages (***: *P* < 0.001, **: *P* < 0.01, *: *P* < 0.05).

**Figure 9:**
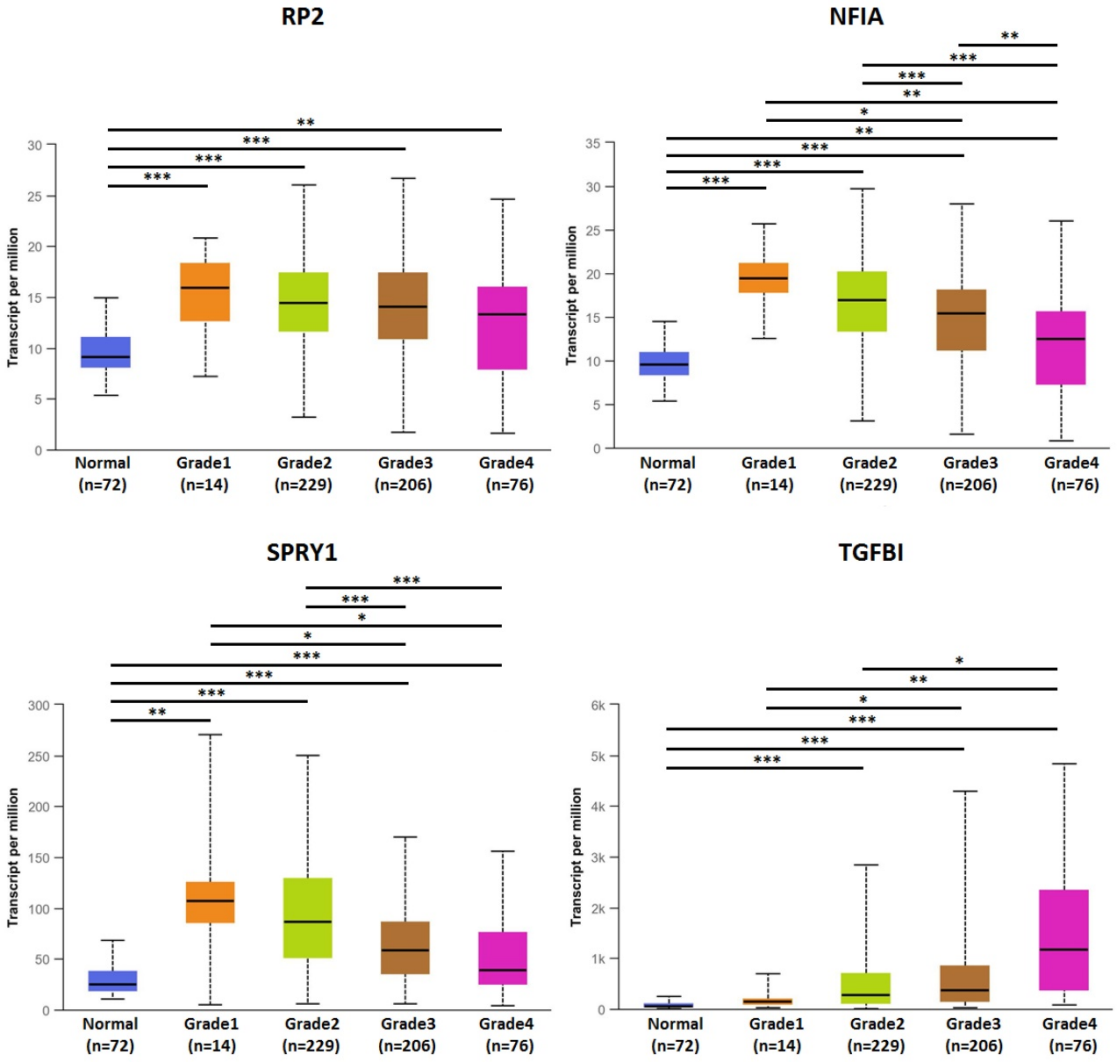
The association of transcriptional expression of hsa-mir-21 predicted target genes (RP2, NFIA, SPRY1 and TGFBI) and KIRC grades (***: *P* < 0.001, **: *P* < 0.01, *: *P* < 0.05).

**Figure 10:**
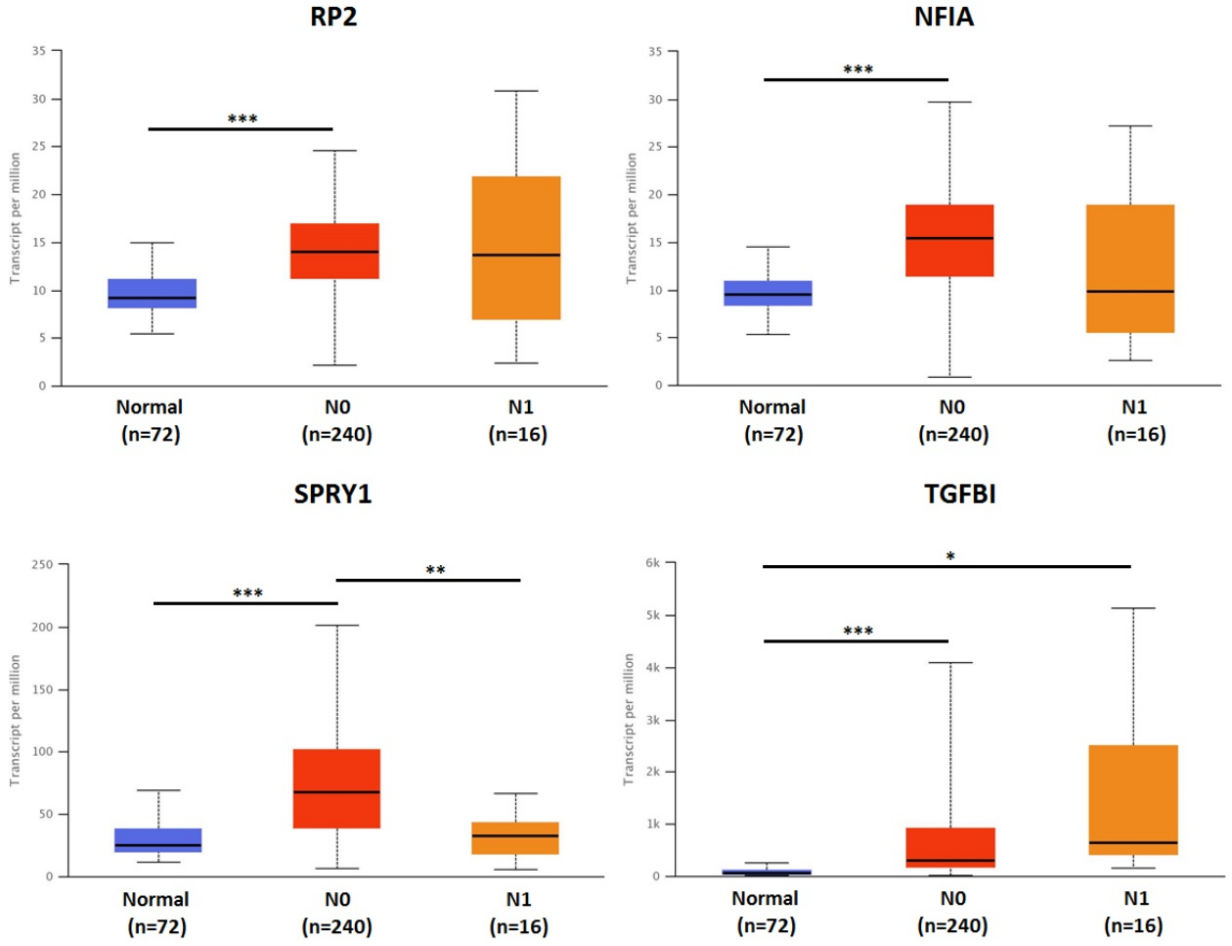
The association of transcriptional expression of hsa-mir-21 predicted target genes (RP2, NFIA, SPRY1 and TGFBI) and KIRC nodal metastasis status (***: *P* < 0.001, **: *P* < 0.01, *: *P* < 0.05).

The association of transcriptional expression of hsa-mir-21 predicted target genes (RP2, NFIA, SPRY1 and TGFBI) and clinicopathological parameters of KIRC patients were analyzed by UALCAN, including KIRC stages, KIRC grades and nodal metastasis status **(Figures 8, 9 and 10)**. Figure 8 indicated that the transcriptional expression of hsa-mir-21 predicted target genes (RP2, NFIA, SPRY1 and TGFBI) were significantly relevant to KIRC stages. The transcriptional expression of RP2, NFIA, SPRY1 and TGFBI in four cancer stages was significantly higher than that of the normal. The highest transcriptional expressions of RP2, NFIA and SPRY1 were found in stage 1. Similarly, Figure 9 indicated that the transcriptional expression of hsa-mir-21 predicted target genes (RP2, NFIA, SPRY1 and TGFBI) were significantly relevant to KIRC grades. The transcriptional expression of RP2, NFIA and SPRY1 in four cancer grades was significantly higher than that of the normal. The highest transcriptional expressions of RP2, NFIA and SPRY1 were found in grade 1. The highest transcriptional expressions of TGFBI were found in grade 4. Figure 10 indicated that the high expression of SPRY1 suggested low risk of lymph node metastasis, whereas the high expression of TGFBI suggested high risk of lymph node metastasis.

### Single cell analysis of hsa-mir-21 predicted target genes (RP2, NFIA, SPRY1 and TGFBI) in normal kidney tissue and immune infiltration level and correlation in KIRC patients

**Figure 11:**
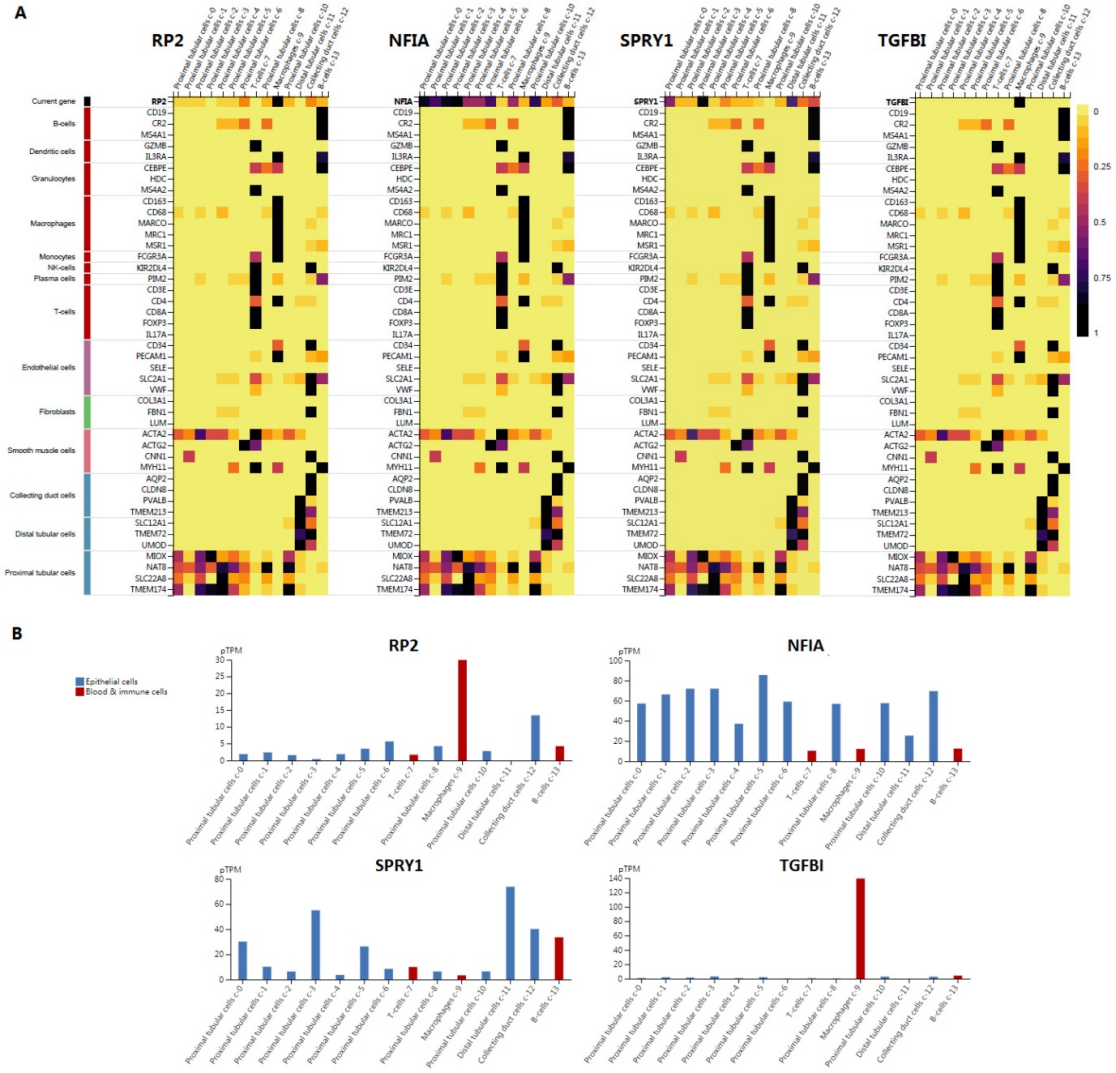
Single cell analysis of hsa-mir-21 predicted target genes (RP2, NFIA, SPRY1 and TGFBI) in normal kidney tissue.

**Figure 12:**
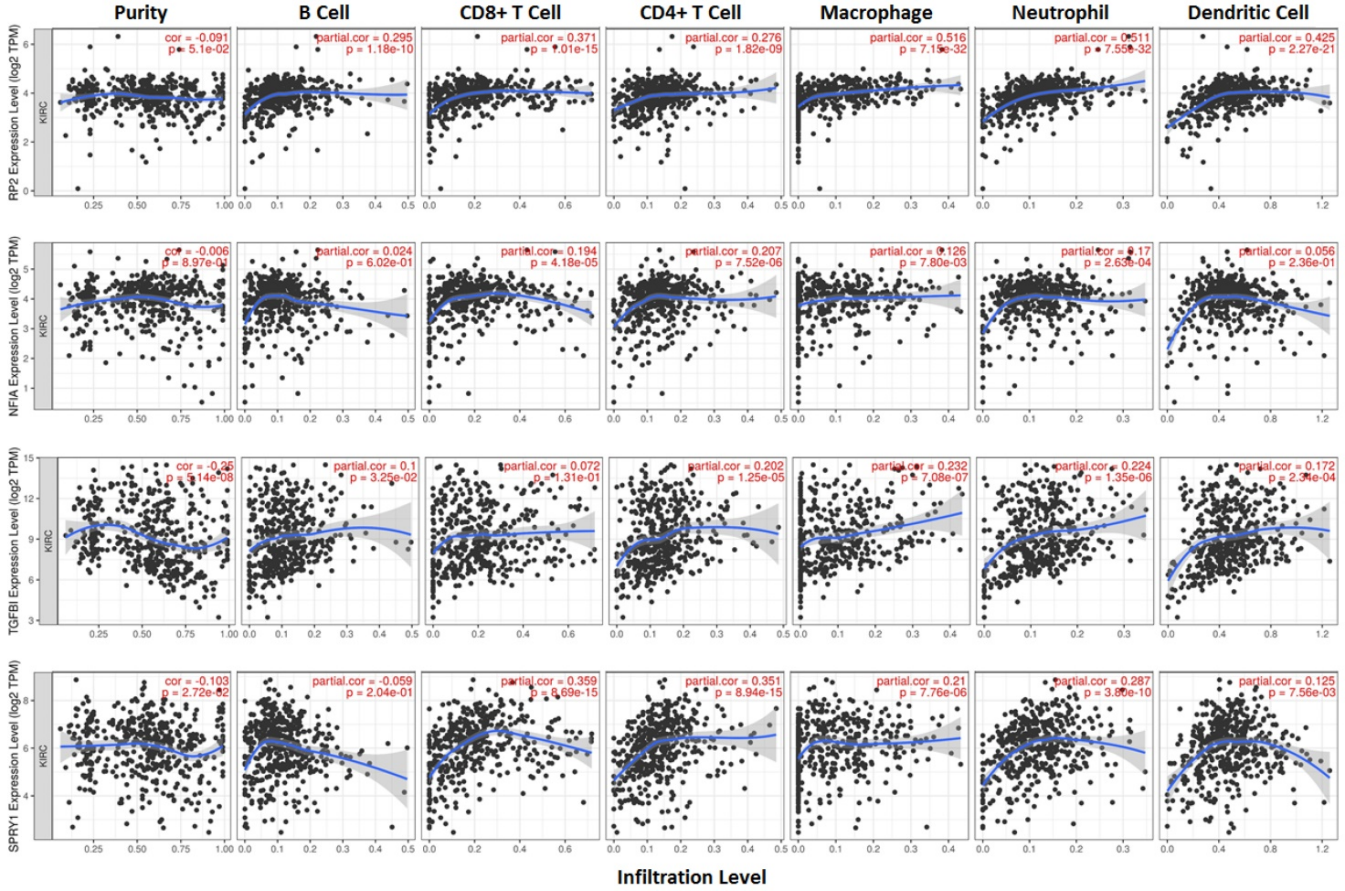
The association of expression of hsa-mir-21 predicted target genes (RP2, NFIA, SPRY1 and TGFBI) and immune infiltration level in KIRC.

**Figure 13:**
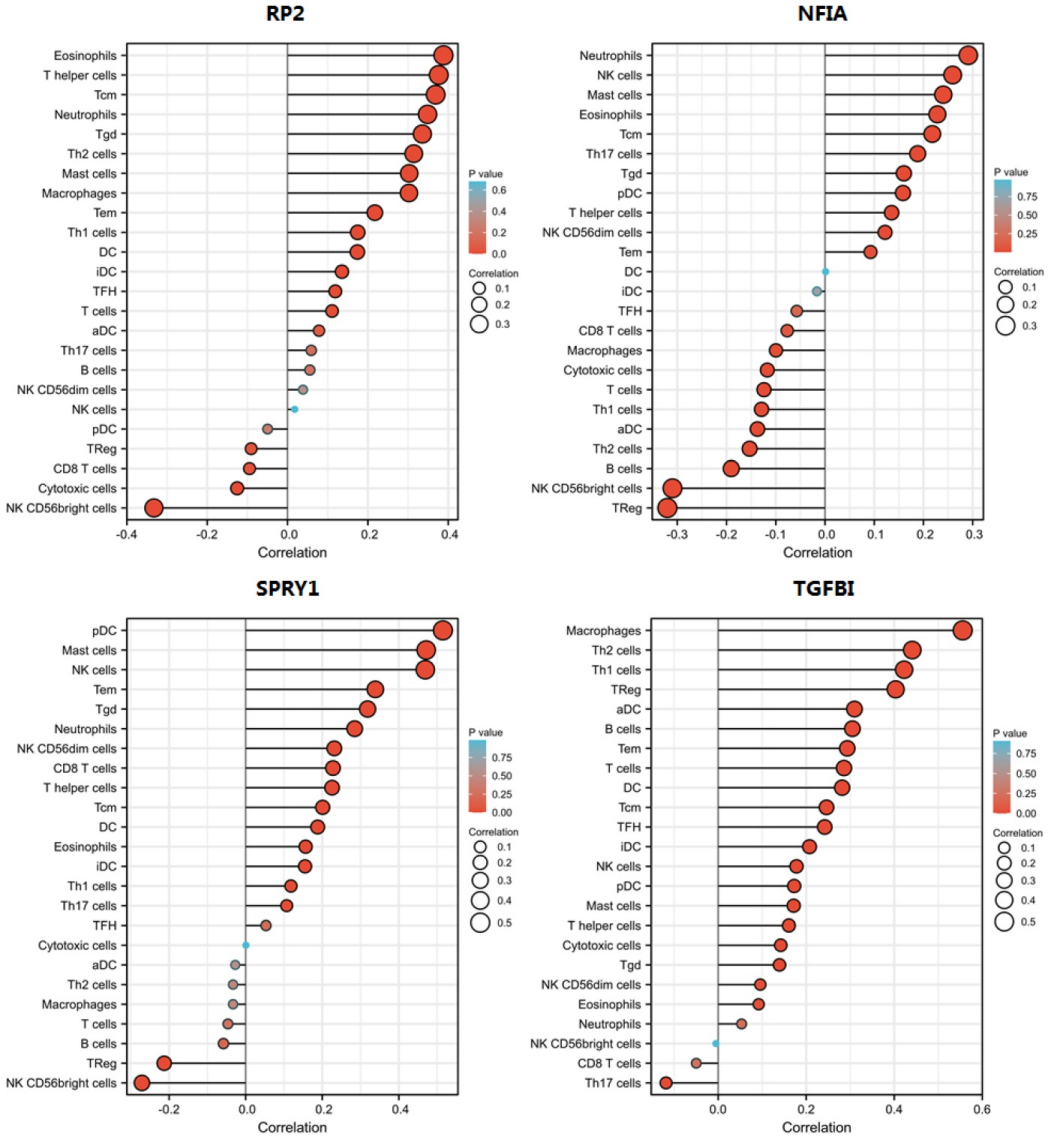
The correlation of expression of hsa-mir-21 predicted target genes (RP2, NFIA, SPRY1 and TGFBI) with immune infiltration in KIRC.

**Figure 14:**
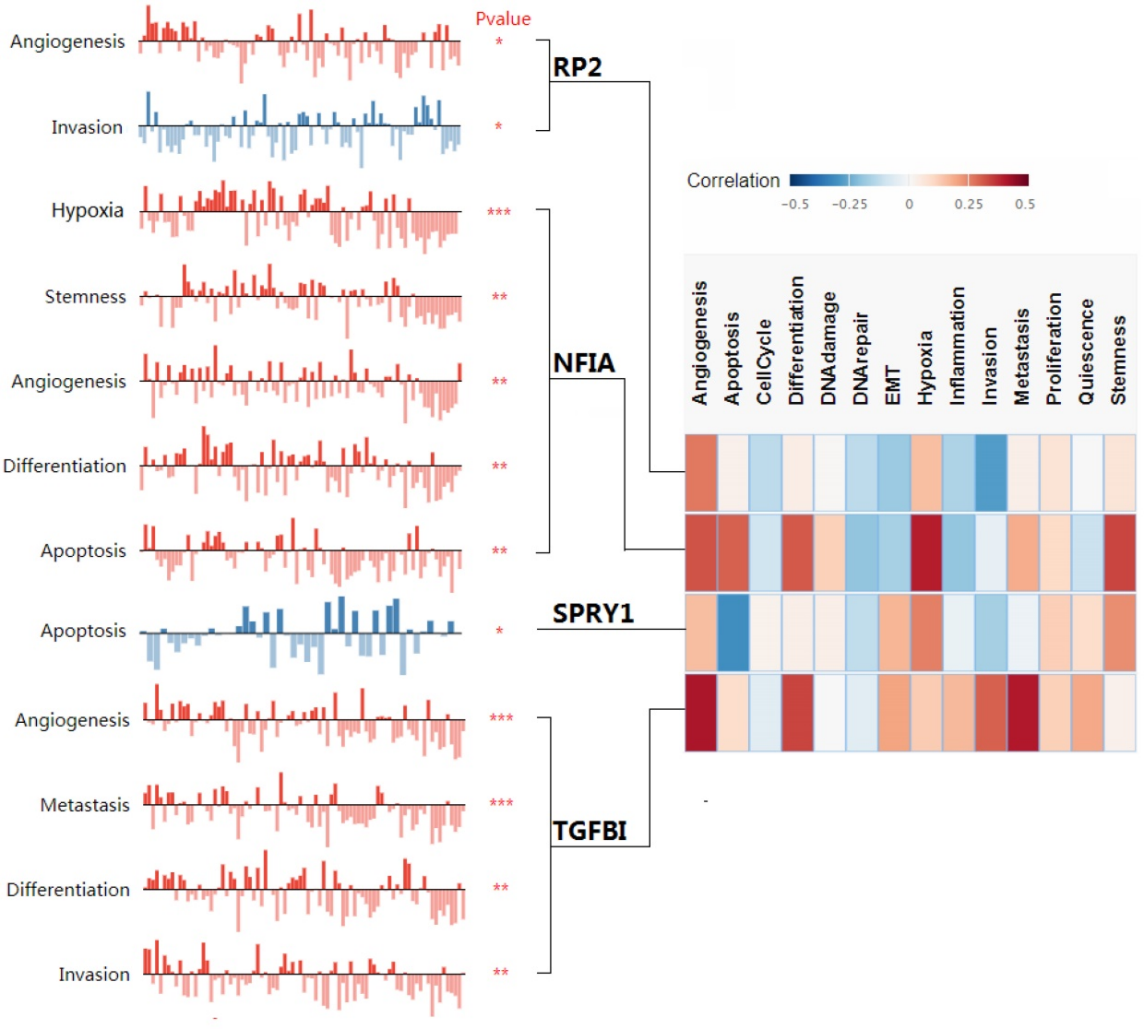
The associated cancer functional states of hsa-mir-21 predicted target genes (RP2, NFIA, SPRY1 and TGFBI).

**Figure 15:**
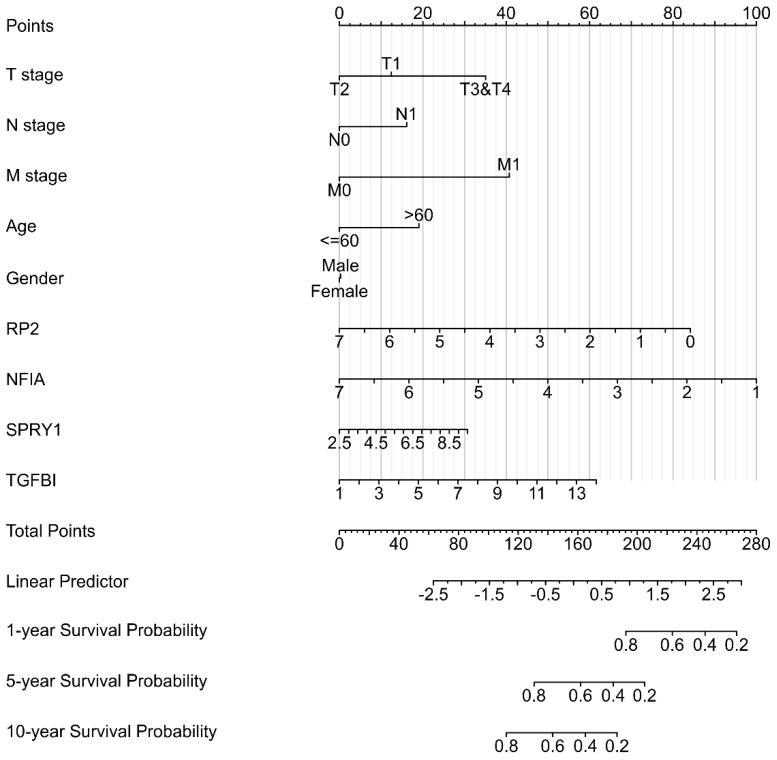
The Nomogram of KIRC,

Cluster cell type of hsa-mir-21 predicted target genes (RP2, NFIA, SPRY1 and TGFBI) in kidney tissue were analyzed by Human Protein Atlas **(Figure 11)**. Figure 11 showed that the highest cluster cell type of RP2, NFIA, SPRY1 and TGFBI in normal kidney tissue were macrophages c-9, proximal tubular cells c-5, distal tubular cells c-11 and macrophages c-9. The association of expression of hsa-mir-21 predicted target genes (RP2, NFIA, SPRY1 and TGFBI) and immune infiltration level in KIRC patients were analyzed by TIMER2.0, including B cell, CD8+ T cell, CD4+ T cell, macrophage, neutrophil and dendritic cell** (Figure 12)**. Figure 12 indicated that the expression of hsa-mir-21 predicted target genes (RP2, NFIA, SPRY1 and TGFBI) were significantly relevant to the immune infiltration level in KIRC. TCGA and R package: GSVA package (version 1.34.0) were used to analyze the correlation of expression of hsa-mir-21 predicted target genes (RP2, NFIA, SPRY1 and TGFBI) and immune infiltration in KIRC patients **(Figure 13, Tables 2, 3, 4 and 5)**. The associated cancer functional states of hsa-mir-21 predicted target genes (RP2, NFIA, SPRY1 and TGFBI) was first revealed **(Figure 14)**. The survival and prognosis model of KIRC was established based on hsa-mir-21 predicted target genes (RP2, NFIA, SPRY1 and TGFBI) expression and related clinical features **(Figure 15)**.

### Transcription factor prediction and DNA base change of hsa-mir-21 predicted target genes (RP2, NFIA, SPRY1 and TGFBI)

**Figure 16:**
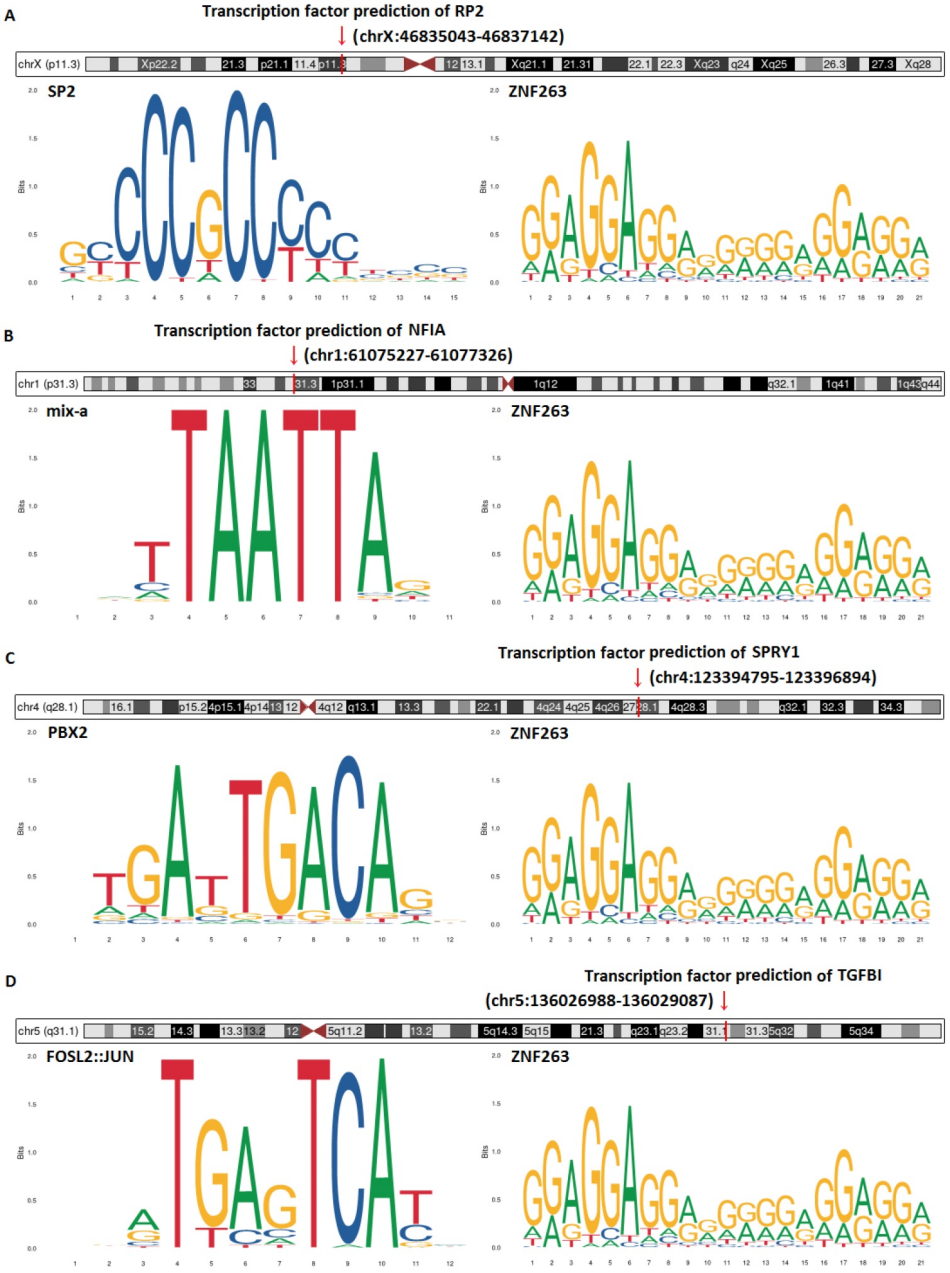
Transcription factor prediction and DNA base change of hsa-mir-21 predicted target genes (RP2, NFIA, SPRY1 and TGFBI).

Transcription factor prediction and DNA base change of hsa-mir-21 predicted target genes (RP2, NFIA, SPRY1 and TGFBI) were analyzed by JASPAR and Gene database (NCBI)** (Figure 16)**. Figure 16 showed that transcription factor prediction of RP2 was in chrX:46835043-46837142, transcription factor prediction of NFIA was in chr1:61075227 -61077326, transcription factor prediction of SPRY1 was in chr4:123394795-123396894 and transcription factor prediction of TGFBI was in chr5:136026988-136029087. The top two transcription factors with the highest scores of RP2 were SP2 and ZNF263. The top two transcription factors with the highest scores of NFIA were mix-a and ZNF263. The top two transcription factors with the highest scores of SPRY1 were PBX2 and ZNF263. The top two transcription factors with the highest scores of TGFBI were FOSL2::JUN and ZNF263. Of note, ZNF263 was their common predictive transcription factor.

### Common predictive transcription factor ZNF263 as an independent indicator for prognosis in KIRC patients

**Figure 17:**
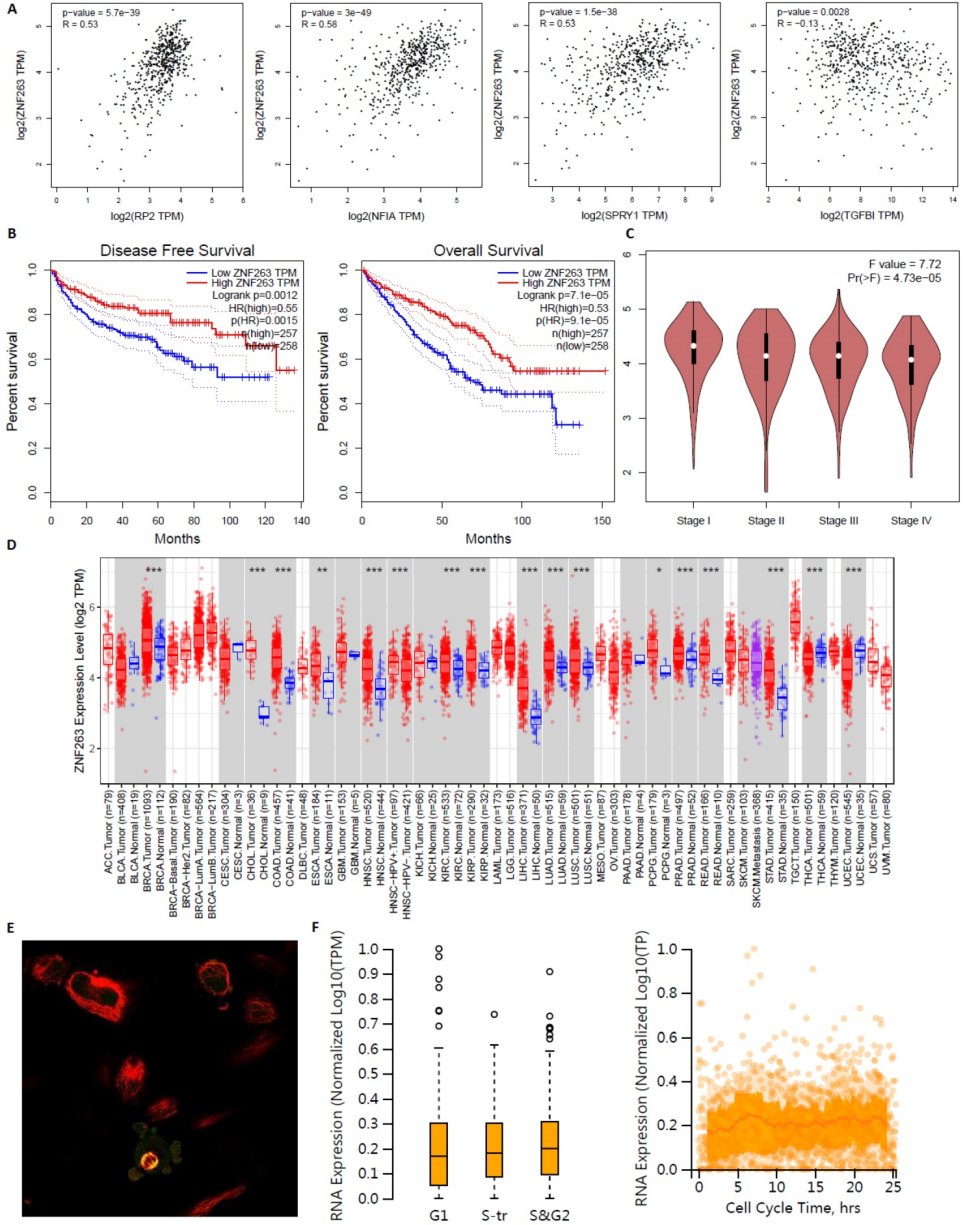
Transcription factor ZNF263 as an independent indicator for prognosis in KIRC patients.

ZNF263 as an independent indicator for prognosis in KIRC patients were analyzed by GEPIA, TIMER2.0 and Human Protein Atlas **(Figure 17)**. Figure 17A verified that the correlation between ZNF263 and RP2 (*P*=5.7e-39), NFIA (*P*=3e-49), SPRY1 (*P*=1.5e-38), TGFBI (*P*=0.0028) were all statistically significant based on Spearman's rank correlation coefficient. Figure 17B showed that the disease free survival and overall survival analysis of the expression of ZNF263 in KIRC. High expression of ZNF263 was a favorable factor for the prognosis of KIRC. Figure 17D showed that a pan-cancer analysis of transcription factor ZNF263. Figure 17E indicated that ZNF263 mainly localized to the cytosol and also localized to the nucleoplasm and mitotic spindle. However, variation in transcript expression of ZNF263 was not correlated to the cell cycle.

## Discussion

KIRC was the most common pathological type of renal cancer and the eighth most common cancer in the world 19. The early clinical symptoms usually occur when the renal tumor volume was large enough. Most patients with KIRC are in the middle and late stage when they seek medical treatment, leading to missing the optimal treatment time. Therefore, the mortality and recurrence rates of KIRC were high 20,21. The occurrence and progression of KIRC were urgently needed to be studied and some biomarkers that can be used for early diagnosis of KIRC should be explored. Generally, miRNAs had their own predicted target genes, and changes in miRNA expression can alter the transcription expression level of miRNAs predicted target genes, which was relevant to the occurrence and development of various cancers 22. Previous studies have reported that hsa-mir-21 was associated with tumorigenesis and tumor metastasis processes 23, the specific role of hsa-mir-21 and its predicted target genes in KIRC remain limited understanding. At present, there is a lack of effective biomarkers for early diagnosis or prognosis assessment of KIRC in clinical work. To our knowledge, this is the first elucidation of the mRNA expression level and prognostic value of hsa-mir-21 and its predicted target genes (RP2, NFIA, SPRY1 and TGFBI) in KIRC. In addition, the upstream study revealed the common key transcription factor ZNF263 of the four genes, and the downstream study described the immune infiltration level and correlation, thus enriching the exploration depth of the mechanism of KIRC genesis and metastasis.

Our research observed that over expression of has-mir-21 and its predicted target genes (TAGAP, FASLG, COL4A1, CD69, MSX1, RP2, PPP1R3B, NFIA, TGFBI, CCL20, SPRY1, PCSK6, S100A10 and MEF2C) were in KIRC. Predicted functional pathways of over-expressed hsa-mir-21 predicted target genes of and other 30 gene partners indicating the potential signaling pathways. Of note, up-transcriptional expressions of RP2, NFIA, SPRY1 were significantly associated with favorable prognosis in KIRC, whereas up-transcriptional expression of TGFBI was markedly significantly to unfavorable prognosis in KIRC. Multivariate clinicopathological analysis indicated that up-transcriptional expression of hsa-mir-21 predicted target genes (RP2, NFIA, SPRY1 and TGFBI) were significantly relevant to KIRC stages and grades. Besides, high expression of SPRY1 suggested low risk of lymph node metastasis, whereas the high expression of TGFBI suggested high risk of lymph node metastasis. In hsa-mir-21 predicted target genes downstream study, expressions of RP2, NFIA, SPRY1 and TGFBI were significantly relevant to the immune infiltration. In hsa-mir-21 predicted target genes upstream study, ZNF263 was a common predicted transcription factor of RP2, NFIA, SPRY1 and TGFBI, which can as an independent indicator for prognosis in KIRC patients.

**RP2**, RP2 activator of ARL3 GTPase, can involve in the transport between the Golgi apparatus and the ciliary membrane as well as protein localization. RP2 mutations can lead to rod-shaped photoreceptor cell death leading to severe X-linked retinitis pigmentosa 24. The results of the latest study suggest that females with RP2 generally maintain good visual acuity throughout their lives, while males affected by RP2 gradually lose vision 25. However, studies on RP2 mutations in tumors have been limited. Hurd et al. demonstrated that RP2 is localized to the primary cilia by amino-terminal diacylation in renal epithelium and the complex of RP2 and polycystic protein was associated with the development of polycystic nephropathy 26. It was not clear how RP2 regulates intra-ciliary polycystic 2 protein transport, but it can be inferred that RP2 was involved in nephron development. To our knowledge, this was the first time that the role of RP2 in KIRC has been explored. Transcriptional expression of RP2 was significantly relevant to KIRC stages, grades, and OS. Cancer single-cell analysis indicated expression of RP2 was related to the Inhibition of cancer Invasion. We found that RP2 was favorable for the KIRC prognosis, which can be a potential biomarker of KIRC prognosis.

**NFIA**, nuclear factor I A, can encode the transcription factor of the NF1 family. Zhu at el. found that miR-671-5p can promote prostate cancer development and invasion via inhibiting the NFIA axis, which indicated that the expression of NFIA was a protective factor in prostate cancer patients 27. Up-regulation of NFIA can slow the development of colorectal cancer through inhibiting cancer cell proliferation and metastasis 28. In addition, NFIA was related to cancer treatment response. Sun at el. demonstrated that NFIA can improve radiotherapy sensitivity in non-small cell lung cancer via attenuating the expression level of p-AKT and p-ERK 29. However, another study suggested that enhanced expression of NFIA was associated significantly with unfavorable cancer cell differentiation and high risk of lymph node metastasis in esophageal squamous cell carcinoma 30. These studies indicated that NFIA may have different roles in different cancers. In our research, the transcriptional expression of NFIA in KIRC was markedly increased, suggesting a good prognosis. However, the key target of NFIA in the role switch between cancer suppression and cancer genesis are unclear at present. Therefore, we speculated that the specificity of cancer immune microenvironment may account for the role switch of NFIA in cancers.

**SPRY1**, sprouty RTK signaling antagonist 1, can participate in the negative feedback of fibroblast growth factor receptor signaling pathway. It was reported that knockdown of SPRY1 can reduce the risk of distant metastasis from triple negative breast cancer via inhibiting EGF/EGFR mediated pathways 31. Lv et al. found that overexpression of SPRY1 can promote cell proliferation and shorten cell cycle in acute myeloid leukemia patients via hedgehog pathway, which suggested a poor prognosis 32. Similarly, Montico et al. reported that the suppression of SPRY1 can cause damage to cancer cells in cutaneous melanoma by blocking cell division cycle and promoting cell apoptosis 33.

However, it has been reported that high expression of SPRY1 can reduce the proliferation, invasion and distant metastasis of ovarian cancer cells 34. In KIRC patients, up-expression of SPRY1 was associated with a good prognosis. Combined with existing studies 33,34 and our findings, we speculate that the function of SPRY1 in KIRC may affect cancer cells by regulating cell cycle, cell proliferation and cell apoptosis.

**TGFBI**, transforming growth factor beta induced, can encode RGD-containing protein, which can modulate cell adhesion. Animal experimental studies have shown that TGFBI plays a key role in inducing breast cancer metastasis and promoting cancer progression by regulating cancer microenvironment and hypoxia 35. Chiavarina et al. found that TGFBI can promote cancer invasion and liver metastasis in colorectal cancer in relation to its ability to stimulate angiogenesis 36. Steitz et al. demonstrated that TGFBI was associated with tumor migration and tumor-free survival in ovarian cancer 37. Existing studies of the expression of TGFBI in urinary tumors also suggest a poor prognosis. Abnormal activation of the TGF-β signaling pathway in benign prostatic epithelium can lead to increased expression of the pro-tumor invasion factor TGFBI, which may contribute to the progression of prostate cancer 38. Over-expression of TGFBI was markedly significantly to poor prognosis in KIRC in our study, which can stimulate the angiogenesis, metastasis, differentiation and invasion in cancer. Thus, TGFBI can as an independent indicator for prognosis in KIRC patients.

## Conclusion

In short, we concluded that hsa-mir-21 predicted target genes (RP2, NFIA, SPRY1 and TGFBI) and the common transcription factor ZNF263 could be the advanced prognosis biomarkers in KIRC patients.

## Supplementary Material

Supplementary table: Target genes prediction of has-mir-21.

## Figures and Tables

**Table 1:** Up-regulated hsa-mir-21 predicted target genes in KIRC

Hsa-mir-21 predicted target genes	Description	Expression in KIRC
TAGAP	T cell activation RhoGTPase activating protein	Up-regulation
FASLG	Fas ligand	Up-regulation
COL4A1	collagen type IV alpha 1 chain	Up-regulation
CD69	CD69 molecule	Up-regulation
MSX1	msh homeobox 1	Up-regulation
RP2	RP2 activator of ARL3 GTPase	Up-regulation
PPP1R3B	protein phosphatase 1 regulatory subunit 3B	Up-regulation
NFIA	nuclear factor I A	Up-regulation
TGFBI	transforming growth factor beta induced	Up-regulation
CCL20	C-C motif chemokine ligand 20	Up-regulation
SPRY1	sprouty RTK signaling antagonist 1	Up-regulation
PCSK6	proprotein convertase subtilisin/kexin type 6	Up-regulation
S100A10	S100 calcium binding protein A10	Up-regulation
MEF2C	myocyte enhancer factor 2C	Up-regulation

**Table 2:** The correlation of RP2 with immune infiltration in KIRC

Gene	Cells	r (Pearson)	*P* value	r (Spearman)	*P* value
RP2	aDC	0.124	0.004	0.078	0.069
RP2	B cells	0.000	0.994	0.056	0.198
RP2	CD8 T cells	-0.082	0.057	-0.095	0.028
RP2	Cytotoxic cells	-0.070	0.105	-0.125	0.004
RP2	DC	0.085	0.050	0.174	<0.001
RP2	Eosinophils	0.392	<0.001	0.388	<0.001
RP2	iDC	0.118	0.006	0.135	0.002
RP2	Macrophages	0.327	<0.001	0.302	<0.001
RP2	Mast cells	0.258	<0.001	0.303	<0.001
RP2	Neutrophils	0.342	<0.001	0.349	<0.001
RP2	NK CD56bright cells	-0.339	<0.001	-0.333	<0.001
RP2	NK CD56dim cells	0.053	0.219	0.039	0.372
RP2	NK cells	-0.014	0.754	0.018	0.680
RP2	pDC	-0.036	0.398	-0.050	0.250
RP2	T cells	0.139	0.001	0.111	0.010
RP2	T helper cells	0.486	<0.001	0.376	<0.001
RP2	Tcm	0.431	<0.001	0.369	<0.001
RP2	Tem	0.244	<0.001	0.217	<0.001
RP2	TFH	0.114	0.008	0.119	0.006
RP2	Tgd	0.281	<0.001	0.336	<0.001
RP2	Th1 cells	0.201	<0.001	0.175	<0.001
RP2	Th17 cells	0.018	0.676	0.059	0.172
RP2	Th2 cells	0.336	<0.001	0.314	<0.001
RP2	TReg	-0.024	0.580	-0.091	0.036

**Table 3:** The correlation of NFIA with immune infiltration in KIRC

Gene	Cells	r (Pearson)	*P* value	r (Spearman)	*P* value
NFIA	aDC	-0.069	0.107	-0.137	0.001
NFIA	B cells	-0.122	0.005	-0.190	<0.001
NFIA	CD8 T cells	-0.079	0.066	-0.077	0.075
NFIA	Cytotoxic cells	0.076	0.078	-0.117	0.006
NFIA	DC	0.016	0.705	0.001	0.978
NFIA	Eosinophils	0.247	<0.001	0.228	<0.001
NFIA	iDC	-0.019	0.662	-0.016	0.705
NFIA	Macrophages	-0.019	0.666	-0.100	0.021
NFIA	Mast cells	0.259	<0.001	0.240	<0.001
NFIA	Neutrophils	0.337	<0.001	0.291	<0.001
NFIA	NK CD56bright cells	-0.262	<0.001	-0.310	<0.001
NFIA	NK CD56dim cells	0.186	<0.001	0.122	0.005
NFIA	NK cells	0.232	<0.001	0.259	<0.001
NFIA	pDC	0.280	<0.001	0.158	<0.001
NFIA	T cells	0.028	0.510	-0.124	0.004
NFIA	T helper cells	0.164	<0.001	0.135	0.002
NFIA	Tcm	0.306	<0.001	0.218	<0.001
NFIA	Tem	0.175	<0.001	0.092	0.032
NFIA	TFH	-0.026	0.543	-0.057	0.184
NFIA	Tgd	0.179	<0.001	0.160	<0.001
NFIA	Th1 cells	0.007	0.871	-0.129	0.003
NFIA	Th17 cells	0.213	<0.001	0.188	<0.001
NFIA	Th2 cells	-0.117	0.007	-0.153	<0.001
NFIA	TReg	-0.180	<0.001	-0.320	<0.001

**Table 4:** The correlation of SPRY1 with immune infiltration in KIRC

Gene	Cells	r (Pearson)	*P* value	r (Spearman)	*P* value
SPRY1	aDC	0.041	0.344	-0.027	0.528
SPRY1	B cells	-0.046	0.282	-0.058	0.178
SPRY1	CD8 T cells	0.229	<0.001	0.228	<0.001
SPRY1	Cytotoxic cells	0.108	0.012	0.001	0.988
SPRY1	DC	0.180	<0.001	0.188	<0.001
SPRY1	Eosinophils	0.181	<0.001	0.157	<0.001
SPRY1	iDC	0.152	<0.001	0.155	<0.001
SPRY1	Macrophages	0.011	0.807	-0.033	0.445
SPRY1	Mast cells	0.474	<0.001	0.471	<0.001
SPRY1	Neutrophils	0.285	<0.001	0.285	<0.001
SPRY1	NK CD56bright cells	-0.255	<0.001	-0.271	<0.001
SPRY1	NK CD56dim cells	0.282	<0.001	0.231	<0.001
SPRY1	NK cells	0.461	<0.001	0.469	<0.001
SPRY1	pDC	0.588	<0.001	0.515	<0.001
SPRY1	T cells	0.021	0.626	-0.046	0.282
SPRY1	T helper cells	0.229	<0.001	0.226	<0.001
SPRY1	Tcm	0.241	<0.001	0.201	<0.001
SPRY1	Tem	0.378	<0.001	0.339	<0.001
SPRY1	TFH	0.053	0.217	0.053	0.219
SPRY1	Tgd	0.289	<0.001	0.318	<0.001
SPRY1	Th1 cells	0.178	<0.001	0.118	0.006
SPRY1	Th17 cells	0.127	0.003	0.107	0.013
SPRY1	Th2 cells	-0.018	0.673	-0.033	0.445
SPRY1	TReg	-0.146	<0.001	-0.213	<0.001

**Table 5:** The correlation of TGFBI with immune infiltration in KIRC

Gene	Cells	r (Pearson)	*P* value	r (Spearman)	*P* value
TGFBI	aDC	0.318	<0.001	0.310	<0.001
TGFBI	B cells	0.292	<0.001	0.305	<0.001
TGFBI	CD8 T cells	-0.041	0.345	-0.050	0.249
TGFBI	Cytotoxic cells	0.195	<0.001	0.142	<0.001
TGFBI	DC	0.272	<0.001	0.282	<0.001
TGFBI	Eosinophils	0.124	0.004	0.092	0.033
TGFBI	iDC	0.200	<0.001	0.208	<0.001
TGFBI	Macrophages	0.546	<0.001	0.556	<0.001
TGFBI	Mast cells	0.177	<0.001	0.172	<0.001
TGFBI	Neutrophils	0.141	0.001	0.053	0.217
TGFBI	NK CD56bright cells	-0.024	0.572	-0.005	0.907
TGFBI	NK CD56dim cells	0.178	<0.001	0.096	0.026
TGFBI	NK cells	0.192	<0.001	0.178	<0.001
TGFBI	pDC	0.175	<0.001	0.173	<0.001
TGFBI	T cells	0.298	<0.001	0.286	<0.001
TGFBI	T helper cells	0.151	<0.001	0.161	<0.001
TGFBI	Tcm	0.231	<0.001	0.246	<0.001
TGFBI	Tem	0.306	<0.001	0.293	<0.001
TGFBI	TFH	0.236	<0.001	0.242	<0.001
TGFBI	Tgd	0.147	<0.001	0.139	0.001
TGFBI	Th1 cells	0.444	<0.001	0.422	<0.001
TGFBI	Th17 cells	-0.126	0.003	-0.118	0.006
TGFBI	Th2 cells	0.427	<0.001	0.441	<0.001
TGFBI	TReg	0.387	<0.001	0.403	<0.001
